# The Negative Predictive Ability of Immature Neutrophils for Bacteremia in Children With Community-Acquired Infections

**DOI:** 10.3389/fped.2020.00208

**Published:** 2020-05-06

**Authors:** Alexandre M. Pimentel, Caroline C. Vilas-Boas, Ticiana S. Vilar, Cristiana M. Nascimento-Carvalho

**Affiliations:** ^1^Bahiana School of Medicine, Bahiana Foundation for Science Development, Salvador, Brazil; ^2^Post-graduation Program in Health Sciences, Federal University of Bahia School of Medicine, Salvador, Brazil; ^3^Department of Pediatrics, Federal University of Bahia School of Medicine, Salvador, Brazil

**Keywords:** bacteremia, children, community-acquired pneumonia, community-acquired infections, immature neutrophils, *Streptococcus pneumoniae*, white blood cell count

## Abstract

**Background:** Bacteremia is a serious condition. We aimed to assess the role of immature neutrophils in peripheral blood smears for prediction of bacteremia in children.

**Methods:** In this cross-sectional study conducted in Salvador, Brazil, blood cultures collected from patients aged ≤18 years were identified. White Blood Cell count (WBC) was performed upon admission. Medical charts were reviewed and cases from the community were included.

**Results:** Out of 833 potentially eligible patients, 263 (31.6%) were excluded. Therefore, the study group comprised 570 patients being blood collected for culture upon admission from all of them and WBC performed upon admission from 566. The median age was 2 years (IQR: 9.4 mo−5 y) and 300 (52.6%) were male. Acute respiratory infection was the most frequent diagnosis (*n* = 388; 68.1%), being 250 (43.9%) lower (LRTI) and 138 (24.2%) upper respiratory tract infections. Blood culture was positive in 9 (1.6%; 95% CI: 0.8–2.9%) cases, out of which 7 (2.8%) had LRTI. *Streptococcus pneumoniae* (*n* = 3), *Haemophilus* (*n* = 2), *Neisseria meningitidis*, viridans streptococci, *Streptococcus agalactiae*, and *Acinetobacter baumanii* (*n* = 1 each) were isolated. The total WBC/mm3 did not differ when children with positive or negative blood culture were compared (12,100 [IQR: 6,950–15,250] vs. 11,000 [IQR: 7,900–14,900]; *P* = 0.9). However, presence of any immature neutrophil was significantly more frequent among patients with bacteremia in comparison with patients without bacteremia (100% [9/9] vs. 40% [223/557]; *P* < 0.001). The absolute number of immature neutrophils was significantly lower among children without bacteremia (0 [IQR: 0–259] vs. 325 [IQR: 275–1,106]; *P* < 0.001). Overall, the area under the ROC curve of the number of immature neutrophils in regard to bacteremia was 0.82 (95% CI: 0.76–0.88; *P* = 0.001). Among 413 patients with absolute number of immature neutrophils <242/mm^3^, none had bacteremia; among 153 patients with absolute number of immature neutrophils ≥242/mm^3^, 9 (5.9%) had bacteremia. Absolute number of immature neutrophils ≥242/mm3 showed: sensitivity 100% (95% CI: 71.7–100%), specificity 74.1% (95% CI: 70.4–77.7%), negative predictive value 100% (95% CI: 99.3–100.0%), and positive predictive value 5.9% (95% CI: 2.9–10.5%). When only children with LRTI were analyzed, the results were similar.

**Conclusion:** The absolute number of immature neutrophils in peripheral blood smear is a potential tool to rule out bacteremia among children with community-acquired infections.

## Introduction

Bacteremia is recognized to be a serious condition in children, when appropriate investigation to guide prompt management is deemed once morbidity and mortality may be significant when the patient is not treated appropriately (1). About four decades ago, children with Fever Without Source (FWS) and peripheral White Blood Cell count (WBC) ≥15,000 cells/mm3 were identified to have high probability of ongoing bacteremia (2). Afterwards, international guidelines started recommending empiric antibiotic administration when a sick child is identified to be at risk of bacteremia (3). However, recent evidence has questioned the accuracy of total WBC as a screen tool for bacteremia in children (4). For instance, in the USA, from 2008 to 2013, different thresholds such as WBC <5,000/mm^3^, WBC ≥15,000/ mm^3^, absolute neutrophil count ≥10,000/ mm^3^, and platelets <100,000/mm^3^ were studied to detect bacteremia among febrile infants and none of these parameters were found to present high accuracy (5). Therefore, specific diagnostic markers in peripheral blood smear are missing. This is particularly important in health care units where only WBC is performed in order to screen serious illnesses.

The implementation of the new polysaccharide conjugate vaccines has contributed for the decrease of bacteremia frequency in children (6). In Brazil, the universal implementation of *Haemophilus influenzae* type b conjugate vaccine occurred in 1999, of pneumococcal 10-valent (PCV10) and meningococcal capsular group C conjugate vaccines occurred in 2010, all targeting children under 2 years of age (7). The vaccine coverage of the PCV10 was considered as high as 89% in the study population at the time of this study, which allows us to suppose that the majority of our cases had been immunized with this vaccine (8). Nevertheless, irrespective of bacteremia rates <1% among children routinely immunized with these conjugate vaccines (9), it is still important to reliably distinguish children who are prone from children who are not prone to have bacteremia. In this scenario, we aimed to assess bacteremia with a true pathogen as well as the role of absolute number of immature neutrophil in peripheral blood smears as a marker for bacteremia in children with community-acquired infections.

## Materials and Methods

### Study Design and Setting

This was a retrospective cross-sectional study conducted at the Pediatric Emergency Room of the Federal University of Bahia Hospital, in Salvador, Brazil, between April 2011 and April 2012. This is a teaching hospital where medical students and residents collect history and examine the patients using standardized forms under the supervision of senior pediatricians and professors from the Federal University of Bahia School of Medicine.

### Selection of Participants

Inclusion criteria comprised community-dwelling children aged ≤18 years who had blood collected for culture upon the first medical evaluation. According to the hospital policy during the study period, blood culture should be performed in every patient with suspicion of an ongoing bacteremic illness. These cases were identified by a daily systematic review of the Bacteriology Laboratory logbook, from Monday to Friday, in order to identify cases from whom blood was collected for culture in the previous day. Each of these cases had the respective medical chart reviewed in order to collect demographic and clinical data which were then registered into pre-defined forms and were used to ensure that blood was collected upon the first medical evaluation of patients from the community. Data were daily retrieved from medical charts by trained doctors (CCV-B and TSV) who also monitored the Bacteriology Laboratory logbook and were blinded to the laboratory results. Immediately after having identified the case as eligible (blood collected for culture upon the first medical evaluation of patients from the community) WBC results were collected from the Laboratory database and registered into a standardized form also on a blinded basis as blood culture results were released always afterwards due to the necessary incubation period. There was no re-analysis of WBC results after blood culture result was available. The information from the Bacteriology Laboratory logbook, medical chart and Laboratory database were collected on the following week-day after patient's admission to hospital. The only information collected 1 week apart was blood culture result. Both doctors used explicit protocol based on which they had been trained. Data collection training occurred during the pilot study, before data was collected for the full-scale research project. During the full-scale research project, the research team had regular meetings every 2 weeks in order to discuss issues that could have arisen during data surveillance and collection. Variables were precisely defined, that is, the definition of each collected variable was known and understood by both doctors who retrieved information so that both doctors collected the same information for each variable. Whenever there was no information in the medical chart, the respective variable was filled with the code for missing variable. Results of other biomarkers were not collected as they were not routinely performed in this teaching hospital and this was a retrospective study.

### Measurements

All patients had WBC performed along with blood culture. WBC was performed in an automatic machine (CELL-DYN Ruby). Whenever any immature neutrophil was found, the test was checked by a technician who read the peripheral blood smear. Absolute immature neutrophils count was computed by multiplying the total WBC by the percentage of immature neutrophils. For blood culture, 0.5–4.0 mL of blood (mean volume 3.1 ± 0.4 mL) were immediately inoculated into 20 mL of supplemented Brain Heart Infusion broth and incubated in Bact/Alert Organon at 35°C for 7 days, according to the manufacturer's instructions. Whenever the Bact/Alert Organon displayed a positive result, the broth was sub-cultured onto Columbia agar with 5% sheep blood and onto chocolate agar at 35°C in a 5% CO_2_ incubator for 18–24 h. Isolated bacteria were identified by using standardized procedures. Pneumococcal serotyping was performed by Multiplex-PCR. The isolates with negative or equivocal results were sent to Adolfo Lutz Institute (National Reference Laboratory, Brazilian Ministry of Health) and subjected to Quellung reaction for definition of capsular serotype.

The final diagnoses were established by the research team after data collection had been finished, based on symptoms, signs, and laboratory tests results retrieved from the medical charts, without considering the diagnoses made by the pediatrician on duty. These diagnoses were initially assigned by a pediatrician, member of the research team (CCV-B) and then they were revised by a senior pediatrician, also member of this research team (CMN-C) when final diagnoses were established. Fever without localizing source was diagnosed when fever was the only finding for up to 7 days of disease. Acute respiratory infection was diagnosed when the patient presented respiratory complaints or auscultatory findings on physical examination, or pulmonary infiltrate/consolidation in the chest radiograph. Lower respiratory tract infection (LRTI) was diagnosed when the patients had tachypnea, crackles, cyanosis, thoracic recession, wheezing in the physical examination, or pulmonary infiltrate/consolidation in the chest radiograph. Patients with acute respiratory infection without criteria fulfillment for LRTI were diagnosed with upper respiratory tract infection. Cellulitis was diagnosed when the child had localized edema plus local inflammatory alterations. Intestinal infection was considered when the family informed diarrhea with fever and/or vomiting. Urinary tract infection was diagnosed when there was fever plus urinary complaints and abnormal urine test which comprised presence of nitrite or WBC ≥5/high power field. From non-toilet trained children, urine was collected by urethral catheterization; from others, midstream urine samples were collected into a sterile jar.

### Outcomes

The primary outcome was bacteremia with a true pathogen. A true pathogen was considered when the blood culture indicated the growth of a pathogenic micro-organism (10).

### Analysis

Data were entered and analyzed into the Statistical Package for Social Sciences (IBM® SPSS Statistics® version 9.0). Categorical variables were presented as absolute and relative (percentage) numbers and continuous variables as median [interquartile range (IQR)]. Categorical variables were compared using the corrected chi square or Fisher exact test and the continuous variables were assessed by Mann Whitney *U*-test, as appropriate. Sensitivity, specificity, positive and negative predictive values were estimated along with the respective 95% confidence interval (95% CI). A receiver operating characteristic (ROC) curve was drawn and the area under the ROC curve (AUC) was calculated along with the respective 95% CI by SPSS version 9.0, taking into account bacteremia as the dependent variable (outcome variable). All tests were 2-tailed with a significance level of 0.05. Cases with missing information were excluded from the analysis of the respective variable. As we studied a convenience consecutive sample of patients, results are presented along with the respective 95% CI in order to express their precision. The study was approved by the Ethics Committee from the Federal University of Bahia (approval number 161.392).

## Results

### Characteristics of Study Subjects

**Figure 1:**
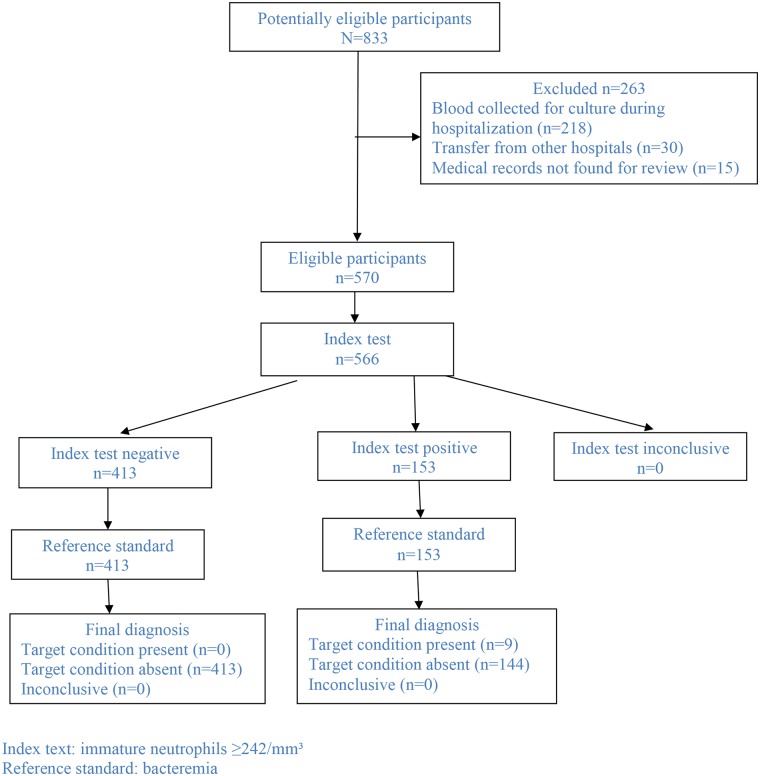
Flowchart of participants through the study.

In the study period, 833 patients aged ≤18 years had blood culture performed, out of which 218 (26.2%) had blood collected for culture during hospitalization (at any time after admission), 30 (3.6%) had been transferred from other hospitals, and 15 (1.8%) did not have their medical records found for review. These 15 patients had negative blood culture. Therefore, this study group comprised 570 patients (Figure 1). Overall, the median age was 2 years (IQR: 9.4 months−5 years) and 300 (52.6%) were male. Acute respiratory infection was the most frequent diagnosis (68.1%; *n* = 388/570), being 43.9% (250/570) LRTI and 24.2% (138/570) upper respiratory tract infections. Other diagnoses were fever without source (*n* = 78/570; 13.7%), cellulitis (*n* = 66/570; 11.6%), intestinal infection (*n* = 23/570; 4.0%), urinary tract infection (*n* = 10/570; 1.8%), and other diagnoses (*n* = 5/570; 0.9%). Table 1 depicts the baseline characteristics of the study group stratified per diagnosis. Overall, 111/570 (19.5%) patients had underlying medical comorbidities such as sickle cell disease (*n* = 35/570; 6.1%), genetic syndrome (*n* = 13/570; 2.3%), neurological disorder (*n* = 12/570; 2.1%), chronic liver disorder (*n* = 11/570; 1.9%), asthma (*n* = 10/570; 1.8%), chronic gastrointestinal disorder (*n* = 7/570; 1.2%), HIV infection (*n* = 5/570; 0.9%), chronic kidney disorder (*n* = 5/570; 0.9%), chronic heart disorder (*n* = 4/570; 0.7%), chronic skin disorder (*n* = 3/570; 0.5%), cystic fibrosis (*n* = 3/570; 0.5%), juvenile rheumatoid arthritis (*n* = 1/570; 0.2%), spherocytosis (*n* = 1/570; 0.2%), and vasculopathy (*n* = 1/570; 0.2%). Patients with co-morbidity were older than patients without it (5.1 [1.9–8.9] vs. 1.7 [0.7–3.9] years, *P* < 0.001).

**Table 1:** Baselines characteristics of the study group stratified per diagnosis.

**Characteristics**	**Frequency *n* (%)^a^**
**LOWER RESPIRATORY TRACT INFECTION (*****n*** **=** **250)**	
Age (median [IQR])	20 [8–54] months
Male gender	135 (54.0)
Fever	207 (82.8)
Cough	204 (81.6)
Difficulty breathing	109 (43.6)
Running nose	80 (32.0)
Nasal blockage	44 (17.6)
Sneezing	15 (6.0)
Cyanosis	10 (4.0)
Tachypnea	139 (62.6)
Wheezing	98 (39.2)
Crackles	97 (38.8)
Chest retraction	62 (24.8)
Lower chest indrawing	42 (16.8)
Grunting	3 (1.2)
Pulmonary infiltrate/consolidation	115 (46.0)
**UPPER RESPIRATORY INFECTION (*****n*** **=** **138)**	
Age (median [IQR])	25 [9–49] months
Male gender	70 (50.7)
Fever	119 (86.2)
Cough	101 (73.2)
Running nose	50 (36.2)
Nasal blockage	32 (23.2)
Sneezing	16 (11.6)
**FEVER WITHOUT SOURCE (*****n*** **=** **78)**	
Age (median [IQR])	37 [10–71] months
Male gender	41 (52.6)
Fever	78 (100.0)
**CELLULITIS (*****n*** **=** **66)**	
Age (median [IQR])	35 [14–92] months
Male gender	31 (47.0)
Local inflammatory alterations	66 (100.0)
Fever	37 (56.1)
**INTESTINAL INFECTION (*****n*** **=** **23)**	
Age (median [IQR])	37 [14–100] months
Male gender	16 (69.6)
Diarrhea	23 (100.0)
Fever	19 (82.6)
Vomiting	12 (52.2)
**URINARY TRACT INFECTION (*****n*** **=** **10)**	
Age (median [IQR])	37 [6–79] months
Male gender	4 (50.0)
Fever	9 (90.0)
Urinary complaint	6 (60.0)
Abnormal urine analysis	10 (100.0)

a *Expressed as absolute number and percentage if not otherwise informed*.

### Main Results

Blood culture was positive in 9 (1.6%; 95% CI: 0.8–2.9%) cases, out of which *Streptococcus pneumoniae* (*n* = 3), *Haemophilus* (*n* = 2), *Neisseria meningitidis*, viridans streptococci, *Streptococcus agalactiae*, and *Acinetobacter baumanii* (*n* = 1, each) were isolated. *A. baumanii* was isolated from a child with fever without source and viridans streptococci were isolated from a child with intestinal infection. None of them had underlying co-morbidity. Viridans streptococci were recovered in pure culture. All other isolated bacteria were recovered from children with LRTI (7/250; 2.8%). The frequency of pneumococcal isolates in this latter group was 1.2% (3/250) and they were serotyped as 3, 6B, and 9L/N. Out of 570 patients, 13 (2.3%) had contaminants in the blood culture, which were identified as coagulase-negative staphylococci. None of these patients were neonates and this contaminant was recovered from just one blood culture. Table 2 shows the recovered bacteria categorized by age groups. There was no age difference when patients with or without bacteremia were compared (1.1 [0.4–5.5] vs. 2.0 [0.8–5.0] years; *P* = 0.7). Bacteremia was more frequent among children with comorbidities, but the difference was not significant (44.4% [49/111] vs. 19.1% [88/459]; *P* = 0.08). No other body fluid culture was performed in the study group.

**Table 2:** Frequency of bacteremia stratified by age group among children with community-acquired infections.

**Age group**	***N***	**Bacteria (% per age group)**
≤ 28 days	8	–
28–90 days	41	*S. agalactiae* (*n* = 1; 2.4%)
3–36 months	295	*S. pneumoniae* (*n* = 2; 0.7%)^a^
		*N. meningitidis* (*n* = 1; 0.3%)
		viridans streptococci (*n* = 1; 0.3%)
>36 months	226	*S. pneumoniae* (*n* = 1; 0.4%)^b^
		*Haemophilus* (*n* = 2; 0.9%)
		*A. baumanii* (*n* = 1; 0.4%)

a *Serotypes: 3, 9 L/N*.

b *Serotypes: 6B*.

**Figure 2:**
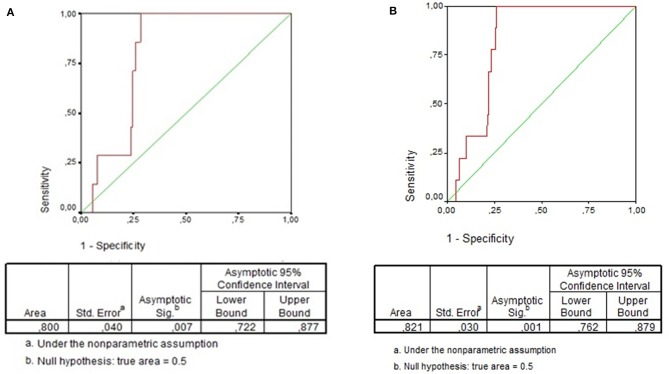
**(A)** ROC Curve of immature neutrophils absolute number for detection of bacteremia in children with community-acquired infections. **(B)** ROC Curve of immature neutrophils absolute number for detection of bacteremia in children with community-acquired lower respiratory tract infection.

The total WBC did not differ when children with positive or negative blood culture were compared (12,100/mm3 [IQR: 6,950–15,250] vs. 11,000/mm3 [IQR: 7,900–14,900]; *P* = 0.9). Overall, total WBC ≥15,000/mm3 was found in 24.6% (139/566) and immature neutrophils were found in 41.0% (232/566) of all cases. However, presence of any immature neutrophil was significantly more frequent among patients with bacteremia in comparison with patients without bacteremia (100% [9/9] vs. 40% [223/557], *P* < 0.001). The same difference was found when this analysis was performed including only children with LRTI (100% [7/7] vs. 44% [106/241], *P* = 0.004). Overall, the absolute number of immature neutrophils was significantly lower among children without bacteremia (median [IQR]: 0/mm3 [0–259] vs. 325/mm3 [275–1,106]; *P* < 0.001). The same result was found when this comparison included only children with LRTI (median [IQR]: 0/mm3 [0–317] vs. 325/mm3 [302–1,442]; *P* = 0.003). Overall, the area under the ROC curve of the number of immature neutrophils in regard to bacteremia was 0.82 (95% Confidence Interval: 0.76–0.88; *P* = 0.001) (Figure 2A). Immature neutrophils absolute number ≥242/mm3 showed sensitivity 100% (95% CI: 71.7–100.0%), specificity 74.1% (95% CI: 70.4–77.7%), negative predictive value 100% (95% CI: 99.3–100.0%), and positive predictive value 5.9% (95% CI: 2.9–10.5%). Figure 1 presents the flowchart with the frequencies of the index test (immature neutrophils absolute number ≥242/mm3) and of the reference standard (bacteremia). As the patient with streptococcus viridans recovered from blood culture had immature neutrophils >242/mm3, when the analysis was repeated after having excluded this case, the same values of sensitivity, specificity, and negative predictive value alongside the respective 95% CI were found and the positive predictive value was 5.3% (95% CI: 2.5–9.7%). When only children with LRTI were analyzed, the area under the ROC curve of the number of immature neutrophils in regard to bacteremia was 0.80 (95% Confidence Interval: 0.72–0.88; *P* = 0.007) (Figure 2B). Sensitivity (100% [95% CI: 65.2–100.0%]), specificity (73.5% [95% CI: 68.9–77.8%]), negative (100% [98.9–100.0%]), and positive (6.5% [2.9–12.5%]) predictive values were similar to the ones reported for the whole study group. Overall, out of 566 patients, 153 (27.0%) had absolute number of immature neutrophils ≥242/mm3 (Figure 1). Out of 566 patients, 139 (24.6%) had total WBC ≥15.000/mm3, and among these 139 cases, 60 (43.2%) had the absolute number of immature neutrophils under 242/mm3.

## Discussion

In this study, the absolute number of immature neutrophils was significantly different when pediatric patients seen with community-acquired infections with or without bacteremia were compared. The high sensitivity value (100% [95% CI: 71.7–100.0%]) is noteworthy. From our results, it is possible to attribute a role to the absolute number of immature neutrophils in screening bacteremia among these patients.

Herein, the median of immature neutrophils absolute number was significantly different when patients with or without bacteremia were compared (325/mm3 vs. 0/mm3; *P* < 0.001). In a study conducted in Turkey, when children aged 3–36 months with fever without source or presumptive viral infection or acute otitis media were included, the same difference was found (619/mm3 vs. 0/mm3) (11). Therein, the area under the ROC curve was 0.785 (95% CI: 0.659–0.911; *P* < 0.001), and the cut-off point of immature neutrophils equal to 194/mm3 showed high negative predictive value (98.6%) and low positive predictive value (16.5%) (9). In our study group, the area under the ROC curve was 0.82 (95% CI: 0.76–0.88; *P* = 0.001) being the cut-off point equal to 242/mm3, with high negative predictive value (100%) and low positive predictive value (5.9%). In spite of the slight difference in the cut-off point with the best performance, it is possible to observe that both studies showed very high negative predictive value of the absolute number of immature neutrophils for bacteremia in children. When we analyzed only children with LRTI, the absolute number of immature neutrophils was also different among patients with or without bacteremia, being the best cut-off point the same and the negative predictive value as high as the one just reported in the total group.

Previous studies have compared the use of procalcitonin and C-reactive protein (CRP) with traditional laboratory tests, such as WBC, in predicting serious bacterial infections, including bacteremia (12–14). A study conducted in Mozambique including children <5 years with severe pneumonia compared the use of procalcitonin and CRP as markers of bacteremia, confirmed with the performance of blood cultures. Therein, the area under the ROC curve in predicting bacteremia was 0.80 for procalcitonin and 0.79 for CRP (13). Mahajan et al. (14) compared the use of procalcitonin with immature neutrophils absolute number, and established cut-off points to predict serious bacterial infections. According to their results, the performance of both tests was quite similar, with negative predictive values of 94% for immature neutrophils absolute number and 93% for procalcitonin (14). Considering the results mentioned above and the ones reported in our study, it is possible to observe that the use of immature neutrophils absolute number is a potential tool for screening children found to be at risk of bacteremia.

On the other side, the total WBC did not differ when patients with or without bacteremia were compared. This finding is similar to results reported by previous studies which demonstrated limited diagnostic value of total WBC for bacteremia (15, 16). According to Herz et al. (9), after the implementation of the polysaccharide conjugate vaccines, the total WBC has become less useful to identify patients at risk of bacteremia. According to our findings, 24.6% of the patients with community-acquired infections were identified as having a WBC ≥15.000/mm3 and, from these patients, 43.2% presented with an absolute number of immature neutrophils under 242/mm3, which means that they were not prone to have bacteremia. Taking into account that 90% of bacteremic children have positive blood culture yield within 24 h after blood incubation (17), it is possible to consider an expectant management when the number of immature neutrophils is under 242/mm^3^.

In a study conducted before the widespread use of the conjugate pneumococcal vaccine, Lee at al. (18) demonstrated that WBC plus selective blood culture and treatment is the least costly approach for children at risk of bacteremia. In a low prevalence setting of bacteremia, after the introduction of the conjugate vaccines against bacteria, the use of the immature neutrophils absolute number could reduce the costs in the initial management, once it is a promising tool to be studied in external validation studies.

In this study, the frequency of bacteremia was low (1.6%) among pediatric patients with community-acquired infections seen in a Pediatric Emergency Room, after the implementation of the available conjugate vaccines in the Region. Our finding is similar to results reported by other studies, which demonstrated a decrease of the frequency of bacteremia to about 1%, after the introduction of the pneumococcal conjugate vaccines (9, 19). However, despite the introduction of the pneumococcal conjugate vaccine, it has been reported that *S. pneumoniae* still remains as the most common causative organism in children presenting with bacteremia (20) and pneumococcal bacteremia still occurs among children seen in the Emergency Room (21). In fact, in our study, *S. pneumoniae* was the most frequent causative organism isolated from children with bacteremia presenting with LRTI (1.2%; 3/250). The frequency of patients diagnosed with urinary tract infection was low (1.8%); that is because, in our teaching hospital, blood culture is rarely performed in patients with such diagnosis, as urine culture is the preferred and standardized bacteriological method of investigation.

This study has potential limitations. This was a retrospective study and the information about the patients was collected from their medical charts. So, the data collection depended on the quality of the information available in the records. However, the study was carried out in a teaching hospital, where procedures and forms are standardized and checked by professors. Notably, LRTI was the diagnosis in almost half of the cases whereas fever without source was the diagnosis in approximately one seventh of the study group. Moreover, 7 (78%) of the 9 isolated bacteria were recovered from patients with LRTI. That is, our results are more appropriate for cases with LRTI than for cases with fever without source. Additionally, the 242/mm^3^ immature neutrophils threshold was not set a priori but data driven and as such it is necessary to be further assessed in external validation studies. The strengths of our work are: the studied sample well represents the patients routinely seen by doctors in daily practice for whom WBC and blood culture are usually ordered and all procedures were standardized according to international standards. Moreover, WBC count is a cheap laboratory test which is feasible to be performed as point-of-care testing. Besides that, with appropriate training, even non-medical, non-healthcare lay users were able to perform WBC count (total and differential) with similar accuracy as trained laboratory professionals when automatic techniques were used, what means that WBC count is an easily reproducible laboratory test (22, 23).

In conclusion, under 242/mm3 immature neutrophils is a potential threshold to rule out bacteremia among children with community-acquired infections, particularly with LRTI, with the advantage of being a cheap laboratory test. Indeed, it is a costless tool as it is always informed in the WBC result.

## Data Availability Statement

The datasets generated for this study are available on request to the corresponding author.

## Ethics Statement

The studies involving human participants were reviewed and approved by Ethics Committee from the Federal University of Bahia (approval number 161.392). Written informed consent from the participants' legal guardian/next of kin was not required to participate in this study in accordance with the national legislation and the institutional requirements.

## Author Contributions

CN-C conceived and designed the study, supervised data collection, and data analysis and interpretation. CV-B and TV collected and entered data, took part in the analysis and interpretation of the results. AP analyzed and interpreted the data and drafted the manuscript. All authors contributed substantially to the manuscript's revision, approved the final version, and agreed to be accountable for all aspects of the work.

## Conflict of Interest

The authors declare that the research was conducted in the absence of any commercial or financial relationships that could be construed as a potential conflict of interest.

## Abbreviations

95% CI: 95% confidence interval
CRP: C reactive protein
FWS: fever without source
IQR: interquartile range
LRTI: lower respiratory tract infections
PCV10: pneumococcal 10-valent conjugate vaccine
ROC: receiver operating characteristic
WBC: white blood cell count.
